# Metagenomic Analysis of Some Potential Nitrogen-Fixing Bacteria in Arable Soils at Different Formation Processes

**DOI:** 10.1007/s00248-016-0837-2

**Published:** 2016-08-31

**Authors:** Agnieszka Wolińska, Agnieszka Kuźniar, Urszula Zielenkiewicz, Artur Banach, Dariusz Izak, Zofia Stępniewska, Mieczysław Błaszczyk

**Affiliations:** 1 Department of Biochemistry and Environmental Chemistry, Institute of Biotechnology, The John Paul II Catholic University of Lublin, 1 I Konstantynów Str, 20-708 Lublin, Poland; 2 Department of Microbial Biochemistry, Institute of Biochemistry and Biophysics PAS, 5a Pawińskiego Str, 02-206 Warsaw, Poland; 3 Department of Microbial Biology, Warsaw University of Life Sciences, Nowoursynowska 159 Str, 02-776 Warsaw, Poland

**Keywords:** Soil metagenomes, Next-generation sequencing, Nitrogen-fixing bacteria, Arable soils, Wastelands

## Abstract

The main goal of the study was to determine the diversity of the potential nitrogen-fixing (PNF) bacteria inhabiting agricultural (A) soils versus wastelands serving as controls (C). The soils were classified into three groups based on the formation process: autogenic soils (*Albic Luvisols*, *Brunic Arenosols*, *Haplic Phaeozem*) formed on loess material, hydrogenic soils (*Mollic Gleysols*, *Eutric Fluvisol*, *Eutric Histosol*) formed under the effect of stagnant water and lithogenic soils (*Rendzina Leptosols*) formed on limestone. In order to determine the preferable conditions for PNF bacteria, the relationships between the soil chemical features and bacterial operational taxonomic units (OTUs) were tested. Additionally, the nitrogen content and fertilisation requirement of the lithogenic (LG), autogenic (AG) and hydrogenic (HG) soils were discussed. The composition of the bacterial communities was analysed with the next-generation sequencing (NGS) by the Ion Torrent™ technology. The sequences were clustered into OTU based on a 99 % similarity threshold. The arable soils tested were distinctly dominated by *β*-*Proteobacteria* representatives of PNF bacteria belonging to the genus *Burkholderia*. Bacteria from the *α*-*Proteobacteria* class and *Devosia* genus were subdominants. A free-living *Cyanobacteria* population dominated in A rather than in C soils. We have found that both soil agricultural management and soil formation processes are the most conducive factors for PNF bacteria, as a majority of these microorganisms inhabit the AG group of soils, whilst the LG soils with the lowest abundance of PNF bacteria revealed the need for additional mineral fertilisation. Our studies have also indicated that there are close relationships between soil classification with respect to soil formation processes and PNF bacteria preference for occupation of soil niches.

**Electronic supplementary material:**

The online version of this article (doi:10.1007/s00248-016-0837-2) contains supplementary material, which is available to authorized users.

## Introduction

Nitrogen (N) is an essential element for microbial and plant life [1, 2]. Mineral N usually comes from three main sources: (a) atmospheric discharges, (b) the biological process of binding and (c) chemical synthesis. All bacteria that possess the capability of molecular N_2_ binding are diazotrophic prokaryotes belonging to two domains: *Bacteria* and *Archaea*. Biological N_2_ fixation is a process of conversion of elemental-unavailable N_2_ into ammonia (NH_4_–N) available to bacteria and plants [2]. In the elemental form, N_2_ can be used only by specialised microorganisms possessing an enzymatic nitrogenase system [3, 4]. A separate group of N-fixing autotrophic bacteria are *Cyanobacteria*, constituting large nitrogen biomass in soils and being responsible for soil fertility [5]. Most soils (especially paddy soils) have a natural population of *Cyanobacteria*, which provides a no-cost potential source of N [6]. The function and diversity of *Cyanobacteria* are well recognised in desert soils [7], saline soils [8], biological soil crust [9] and rice paddy soils [6]. However, knowledge concerning their abundance in agricultural soils [10] and wastelands still remains limited.

The quantity of biologically fixed N_2_ is estimated at c.a. 2 × 10^13^ g N/year [11]. Two modes of molecular N_2_ binding have been identified [12]: (a) symbiotic (*Rhizobia*, *Frankia*), usually amounting to c.a. 150–300 kg N/ha, and (b) non-symbiotic (bacteria, endophytes, lichens, *Cyanobacteria*) ranging from 1 to 20 kg N/ha. Due to the absence of symbiotic bacteria, non-symbiotic N fixation is dominant in many ecosystems, i.e. in green areas of temperate zones, tropical evergreen forests, or deserts [12, 13]. The global rate of N fixation (symbiotic + non-symbiotic) in natural ecosystems may provide 100 × 10^12^ g N/year, which constitutes c.a. 10 % of the annual plant demand. The average content of mineral nitrogen in the Polish soils is in the range of 76–90 kg N/ha in spring and 89–97 kg N/ha in autumn [14, 15]. Dresler et al. [16] found that application of *N*-fertiliser above 121 kg N/ha resulted in a significant increase in the NO_3_–N content in the surface soil layer. According to the Polish Statistical Office report [17], the use of mineral fertilisers in Lubelskie voivodeship amounted to 141.7 kg/ha, with nitrogen, phosphorus and potassium fertilisers accounting for 50, 20 and 38 %, respectively.

There is a relatively long list of symbiotic nitrogen-fixing bacteria. The most common symbiotic N_2_-binding bacteria present in the nodules are able to colonise the rhizosphere and infect legumes. They are classified as slow-growing *Bradyrhizobium* and fast-growing *Rhizobium* [2, 18]. In recent years, a number of the following N-fixing bacteria capable of forming nodules have been isolated and classified into *α*- and *β*-*Proteobacteria*: *Methylobacterium nodulans* [3, 19], *Blastobacter denitrificans* [20], *Devosia neptuniae*, *Devosia riboflavina* and *Devosia natans* [19, 20], *Ochrobactrum lupini* [21], *Agrobacterium* spp. [19], *Azospirillum* spp. [22], *Herbaspirillum lustianum* [23], *Cupriavidus taiwanensis*—recently known as *Ralstonia taiwanensis* [19], *Burkholderia tuberum*, *Burkholderia phymatum* and *Burkholderia cepacia* [3, 22, 24], several strains of γ-*Proteobacteria* [25] and *δ-Proteobacteria* [3].

The best-known and well-recognised processes of N_2_ fixation have been described for *Rhizobia* and legumes, i.e. peas, cow peas, beans and soybeans [2, 26]. The root nodules of *Rhizobia* could reduce even c.a. 20 million tons of atmospheric N_2_ into NH_4_–N, amounting to c.a. 65 % of N utilised in agriculture [2, 26]. However, little is known about other non-symbiotic soil bacteria inhabiting arable soils and having potential for biological N_2_ fixation. Therefore, we have tried to provide new knowledge about the biodiversity of potential nitrogen-fixing (PNF) bacteria in arable soils of different genetic origins. For this purpose, next-generation sequencing (NGS) technique was applied whereby it became possible to omit the inefficient laboratory culture step and acquire knowledge about the enormous microbial groups termed as viable but not cultivable (VBNC). Till now, metagenomic tools with respect to PNF bacteria were applied into the following samples: temperate, subtropical and tropical soils from India [27], Arabian sea oxygen zone [28], Dexing copper mine in China [29] and the experimental station of Embrapa Soja in Brazil [30]. In that context, our study is the first one where PNF bacteria were identified in arable and wasteland sites by NGS Ion Torrent™ technology.

The aim of the study was to recognise the diversity of the PNF soil bacteria and *Cyanobacteria* population living in seven types of arable soils (*Albic Luvisols*, *Brunic Arenosols*, *Haplic Phaeozem*, *Mollic Gleysols*, *Eutric Fluvisol*, *Eutric Histosol*, *Rendzina Leptosols*) versus non-cultivated soils (wastelands) classified into three groups according to the soil formation processes: autogenic (AG), hydrogenic (HG) and lithogenic (LG) soils. Also, correlations between the numbers of operational taxonomic units (OTUs) assigned to the potential N-fixing bacteria and soil chemical variables were assessed. Furthermore, we presented the novelty of ecological studies by indication of relationships between soil science classification with respect to soil formation processes and the number of PNF bacterial OTU number in the three groups of soils.

## Methods

### Experimental Sites

**Fig. 1:**
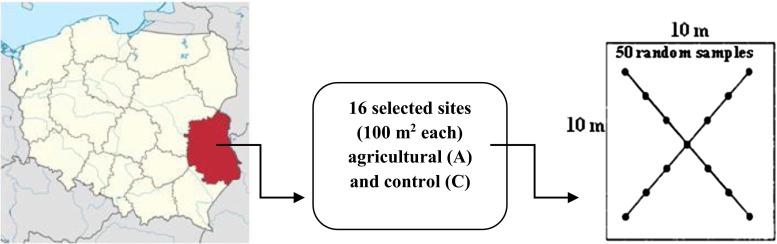
Location of the study site in Lubelskie voivodeship within Poland (according to https://en.wikipedia.org/wiki/Lublin_Voivodeship) with the scheme of soil sampling (according to PN-R-04031:1997) from 16 locations of the Bank of Soil Samples

The study site was located in the south-eastern part of Poland in Lubelskie voivodeship (51° 13′ N, 22° 54′ E), as presented in Fig. 1. The limitation of the study site to one region was prompted by the fact that the Lubelskie voivodeship is characterised by a great diversity of soil types (all basic and Polish dominant soil units are represented here) and is one of the largest and most important agricultural areas in Poland. Farmlands occupy a total of 68 % of Lubelskie voivodeship area, including arable lands up to 78.4 %, meadows and pastures 19.6 % and orchards 2 %. Thirty-one soil units were studied; they were represented by the following soil types (FAO): *Albic Luvisol* (three samples, numbered 1–3), *Brunic Arenosol* (six samples, numbered 4–9), *Haplic Phaezoem* (one sample, numbered 10), *Mollic Gleysol* (two samples, numbered 11–12), *Eutric Fluvisol* (one sample, numbered 13), *Eutric Histosol* (one sample, numbered 14) and *Rendzina Leptosol* (two samples, numbered 15–16) of agricultural (coded A) and wastelands—controls (coded C) were studied (Table 1).

**Table 1:** Description of agricultural soils (Lubelskie voivodeship)

Soil no.	Soil group (field code)	Soil type (FAO)	Geographic coordinates	Crop
1	Autogenic (1–10)	*Albic Luvisol*	22° 10′ 17.7″, 51° 26′ 24.6″	Oat
2		*Albic Luvisol*	22° 27′ 10.3″, 51° 24′ 3.8″	Triticale
3		*Albic Luvisol*	22° 36′ 51.8″, 51° 21′ 27.0″	Wheat
4		*Brunic Arenosol*	22° 06′ 54.2″, 51° 21′ 52.2″	Triticale
5		*Brunic Arenosol*	22° 15′ 19.0″, 51° 23′ 0.9″	Oat
6		*Brunic Arenosol*	22° 15′ 55.5″, 51° 23′ 1.9″	Oat
7		*Brunic Arenosol*	24° 04′ 0.3″, 50° 51′ 15.81	Field prepared for seeding
8		*Brunic Arenosol*	23° 22′ 52.4″, 50° 51′ 14.8″	Triticale
9		*Brunic Arenosol*	22° 07′ 29.9″, 51° 25′ 5.5″	Strawberries
10		*Haplic Phaeozem*	23° 42′ 56.6″, 50° 44′ 48.3″	Triticale
11	Hydrogenic (11–14)	*Mollic Gleysol*	22° 06′ 18.8″, 51° 22′ 48.0″	Colza
12		*Mollic Gleysol*	22° 01′ 25.5″, 51° 29′ 15.3″	Wheat
13		*Eutric Fluvisol*	21° 59′ 10.1″, 51° 33′ 47.7″	Oat
14		*Eutric Histosol*	22° 16′ 38,9″ 51° 25′ 27,3″	Oat
15	Lithogenic (15–16)	*Rendzina Leptosol*	23° 10′ 58.3″ 51° 12′ 22.3″	Celeries
16		*Rendzina Leptosol*	23° 11′ 43.9″ 51° 12′ 10,8″	Oat

Additionally, taking into account the soil’s origin, the investigated material were classified into the three basic groups: autogenic—formed from loess material, represented by *Albic Luvisols* (AL), *Brunic Arenosols* (BA) and *Haplic Phaeozem* (HP), hydrogenic—formed under the influence of stagnant water, represented by *Mollic Gleysols* (MG), *Eutric Fluvisol* (EF) and *Eutric Histosol* (EH) and lithogenic—formed from limestone, represented by *Rendzina Leptosols* (RL). BA and AL cover c.a. 82 % of Poland, hence the highest representativeness of these soil types in our soil collection (9 units of the 16 investigated ones). Soil material and sampling points were carefully selected on the basis of earlier work performed for typological soil recognition in 1991 within the framework of creation of the Bank of Soil Samples (BSS) creation by researchers from the Institute of Agrophysics, Polish Academy of Science in Lublin and the Institute of Land Reclamation and Grassland Farming in Falenty [31]. As an effect of this collaboration, a database for Polish mineral arable soils was created [32]. Given the precise description of the sampling points in the BSS database (name of place and geographic coordinates), there is a possibility of precise returning to the sampling sites [31].

Squares at 10 × 10 m were chosen from each of the 16 sampling points catalogued in BSS database of Lubelskie voivodeship (Fig. 1). Within each square, approximately 50 random soil samples (c.a. 2 kg) were taken from the surface layer (0–20 cm), strictly according to the sampling rules described in the Polish Norm [33]. Wasteland sites, belonging to the same soil type as agricultural lands and located in the nearest neighbourhood to arable soils served as controls. Random samples were combined into one sample in order to obtain the most representative soil material for each investigated site. In this manner, 16 samples were obtained for the agricultural (A) soils and 15 for the controls (C). Due to the close neighbourhood, the same soil, and crop type, soils (BA) numbered 5 and 6 have one control.

### Soil Characteristics

The agricultural and control soils were sampled during the early spring season before plant vegetation and fertilisation (April 2014). An air temperature during sampling amounted to 20 °C; however, an average annual temperature for Lubelskie voivodeship usually does not exceed 10 °C (c.a. 7.3 °C), whereas an average annual rainfall amount to c.a. 560 mm. Under laboratory conditions, each sample was passed through a 2.0-mm sieve and shortly stored at 4 °C prior to the analysis.

Particle size distribution (PSD) was measured using a laser diffractometer Mastersizer 2000 (Malvern, UK) with Hydro G dispersion units [34, 35]. The soils were dispersed using ultrasound at 35 W for 4 min without removing the organic matter [34]. The measurements were carried out in three replications. PSD in the soils investigated, taking into account both the World Reference Base for soil resources (WRB) and the Polish Society of Soil Science (PSSS) classifications, are presented in Table 2. Soil moisture (MOIST) was determined by a gravimetric method (24 h, 105 °C).

**Table 2:** Clay, silt and sand fractions (in volume percentage) obtained by Hydro G unit of laser diffractometer Mastersizer 2000

Sample no.	Loam (mm)	Silt (mm)	Sand (mm)	Particle size group	
	<0.002	0.002–0.05	0.05–2.0	WRB	PSSS
1	4.76	37.66	57.58	Sandy loam	Sandy loam
2	1.25	17.28	81.47	Sandy loam	Sandy loam
3	4.12	55.99	39.88	Silt loam	Loamy silt
4	5.60	50.80	43.59	Silt loam	Loamy silt
5	2.06	22.96	74.98	Sandy loam	Sandy loam
6	3.64	30.88	65.47	Sandy loam	Sandy loam
7	6.43	77.34	16.23	Silt loam	Loamy silt
8	5.26	74.37	20.37	Silt loam	Loamy silt
9	3.69	39.07	57.24	Sandy loam	Sandy loam
10	5.26	77.14	17.60	Silt loam	Loamy silt
11	8.00	79.68	12.32	Silt loam	Loamy silt
12	7.18	32.3	60.52	Sandy loam	Sandy loam
13	2.35	34.50	63.15	Sandy loam	Sandy loam
14	1.74	35.05	63.20	Sandy loam	Sandy loam
15	8.53	65.86	25.61	Silt loam	Loamy silt
16	5.89	66.75	27.36	Silt loam	Loamy silt

*WRB* World Reference Base for soil resources; *PSSS* Polish Society of Soil Science

The soil acidity (pH) and electric conductivity (EC) were determined in triplicate from a 2:1 soil suspension in distilled water using a multifunctional potential metre (Hach Lange, Poland).

Easily degradable carbon (EDC), i.e. a measure of active forms of carbon available for microorganisms and plant roots, was determined in triplicate with the use of UV-1800 (Shimadzu) spectrophotometer (*λ* = 550 nm), by KMnO_4_ digestion and expressed as milligrammes per kilogramme [35, 36].

The concentrations of nitrogen forms (NH_4_–N, NO_3_–N, NO_2_–N) were measured colorimetrically using an AutoAnalyser 3 System (Bran+Luebbe, Germany), according to the description by Wolińska et al. [37]. Each of the measurements was done in triplicate.

More details about the investigated soils and other physicochemical and biological factors are available in our previous studies [35, 37–39].

### DNA Extraction

DNA was extracted within 24 h after sample collection according to the modified procedure elaborated for soil material as described by Tomczyk-Żak et al. [40]. The modification included an additional purification step by CsCl gradient centrifugation (16 h, 70,000 rpm, 20 °C; Sorvall WX Ultra ThermoScietific). More details about the DNA isolation procedure are available in Wolińska et al. [35]. The concentrations of the isolated DNA were quantified with a NanoDrop spectrophotometer (ThermoScientific) after 10-fold dilution, in triplicate.

### Next-Generation Sequencing

To classify soil bacterial communities, amplification of the 16S ribosomal RNA (rRNA) V3 region gene was carried out (27f, 518r). PCR conditions were as follows: 95 °C for 3 min, 30 cycles of 95 °C for 30 s, 53 °C for 30 s and 72 °C for 1 min, with a final extension at 72 °C for 7 min. NGS of the metagenomic16S rRNA amplicons was performed with application of the Ion Torrent™ technology (Ion PGM™, Life Technologies). Amplicons were analysed using recommended kits (Ion Plus Fragment Library Kit, RT-PCR Ion Universal Library Quantitation Kit, Ion PGM™ Template OT2 400 Kit, Qubit™Fluorometric Quantitation). The sequencing step (Ion 318™ Chip Kit v2) was realised in the Laboratory of Microarray Analyses (IBB PAS, Warsaw) according to manufacturer’s instructions.

### Bioinformatics and Statistical Analyses

DNA sequencing data were analysed using MOTHUR v.1.34.4. [41]. The reads were dereplicated and aligned to the MOTHUR-formatted version of the Silva reference database (silva.nr_v119), as described by Quast et al. [42]. Chimeras were detected and removed using UCHIME implementation [43]. The sequences were clustered into OTUs based on a 99 % similarity threshold. A total of 358,289 bp bacterial sequences (for the V3 region) with an average read length of 154 bp were generated across all samples, representing 18,870 OTUs. The taxonomical composition was presented on the interactive Krona Charts [44] based on Table 4 with the number of OTUs.

Additionally, all collected data were statistically processed by means of Statistica 9 PL (StatSoft, USA). The assumptions of parametric tests were checked with Shapiro-Wilk *W* statistics and, if the assumptions were not met, ln(*x* + 1) transformation was applied. The relationships between the numbers of OTUs and physicochemical variables were assessed by means of analysis of regression. First, the correlation matrix was constructed and followed by selection of significant correlations (*p* < 0.05). For the significant correlations, either Pearson’s *r* or Spearman’s rho correlation coefficients were calculated depending on data normality.

## Results

### Physicochemical Soil Properties

Soil texture plays a key role in carbon storage and influences nutrient availability for microorganisms, thus PSD is one of the most important soil parameter crucial for microbiological activity. By comparison of the content of particular fractions, the tested soils were classified into two groups: sandy loam and silt loam/loamy silt (Table 2), due to the dominance of coarser fractions (silt and sand). The chemical characteristic of the soils are shown in Table 3. As shown, there are differences in the chemical features among the C and A sites. Arable soils, at the moment of sampling were characterised by usually lower moisture content (5.2–24.7 %) than control soils (7.1–31.03 %). Moreover, A soils posses acidic pH and by c.a. 22–45 % lower EDC content, compared with the C soils, where higher pH was close to neutral and higher EDC amounts ranging from 575.4 to 1209 mg/kg were available for microorganisms. Taking into account EC and indirect salinity, it was found that the A soils had a higher EC level (0.025–0.168 mS/cm^3^) than the C soils (0.020–0.080 mS/cm^3^). However, for both sites, the EC value did not exceed 2 mS/cm^3^, which classifies the investigated soils in the low saline category. In the case of nitrogen, the nitrate form (NO_3_–N) was dominant both in the C as A soils; however, its concentration was substantially higher in the agricultural soils (2.99–77.2 mg/kg), which resulted from fertilisation, than in the wastelands (1.7–13.8 mg/kg). Additionally, the C soils were characterised by higher ammonia nitrogen content (0.02–4.94 mg/kg) and nitrite nitrogen (0.09–0.87 mg/kg) in contrast to the A soils, where these N forms amounted to 0.01–0.43 and 0.04–0.12 mg/kg, respectively.

**Table 3:** Chemical soil features with respect to control (C) and agricultural (A) soils from Lubelskie voivodeship (±SD)

	MOIST (%)	pH (H_2_O)	EC (mS/cm^3^)	EDC (mg/kg)	NH_4_–N (mg/kg)	NO_3_–N (mg/kg)	NO_2_–N (mg/kg)
C soils							
1C	9.76 ± 0.11	6.27 ± 0.005	0.034 ± 0.003	716.60 ± 0.001	0.09 ± 0.006	1.68 ± 0.014	0.17 ± 0.001
2C	11.16 ± 0.11	5.020 ± 02	0.029 ± 0.002	576.52 ± 1.90	0.04 ± 0.014	5.84 ± 0.03	0.10 ± 0.001
3C	9.13 ± 0.05	6.22 ± 0.09	0.054 ± 0.005	799.78 ± 1.90	0.06 ± 0.006	3.58 ± 0.09	0.42 ± 0.005
4C	13.50 ± 0.10	7.08 ± 0.06	0.062 ± 0.002	947.52 ± 1.90	0.48 ± 0.008	7.57 ± 0.32	0.53 ± 0.003
5C	8.63 ± 0.15	5.58 ± 0.04	0.049 ± 0.001	828.23 ± 0.002	0.69 ± 0.009	10.18 ± 0.14	0.21 ± 0.002
6C	8.63 ± 0.15	5.58 ± 0.04	0.049 ± 0.001	828.23 ± 0.002	0.69 ± 0.009	10.18 ± 0.14	0.21 ± 0.002
7C	12.76 ± 0.11	6.99 ± 0.03	0.065 ± 0.001	1113.9 ± 0.001	0.41 ± 0.008	5.41 ± 0.14	0.87 ± 0.003
8C	20.26 ± 0.63	6.06 ± 0.009	0.058 ± 0.006	919.07 ± 1.90	2.61 ± 0.04	11.07 ± 0.05	0.24 ± 0.02
9C	7.10 ± 0.17	5.40 ± 0.006	0.080 ± 0.001	575.42 ± 0.001	0.18 ± 0.001	1.76 ± 0.06	0.80 ± 0.002
10C	31.03 ± 0.23	7.22 ± 0.02	0.059 ± 0.006	1209.0 ± 3.28	0.02 ± 0.002	8.23 ± 0.02	0.44 ± 0.006
11C	14.33 ± 0.57	6.76 ± 0.01	0.040 ± 0.002	1051.5 ± 3.28	0.78 ± 0.01	10.06 ± 0.09	0.15 ± 0.001
12C	10.40 ± 0.17	6.25 ± 0.03	0.044 ± 0.002	795.40 ± 0.001	4.94 ± 0.008	6.75 ± 0.05	0.10 ± 0.001
13C	8.86 ± 0.11	5.64 ± 0.06	0.025 ± 0.001	620.29 ± 1.90	0.27 ± 0.03	2.20 ± 0.05	0.13 ± 0.002
14C	9.30 ± 0.20	5.27 ± 0.01	0.020 ± 0.001	877.48 ± 0.001	0.02 ± 0.002	9.05 ± 0.03	0.09 ± 0.001
15C	12.50 ± 0.17	5.76 ± 0.01	0.040 ± 0.002	693.62 ± 0.001	3.39 ± 0.06	10.12 ± 0.07	0.09 ± 0.004
16C	19.30 ± 0.17	7.39 ± 0.02	0.070 ± 0.001	1104.0 ± 0.001	0.28 ± 0.02	13.82 ± 0.5	0.13 ± 0.004
A soils							
1A	8.20 ± 0.20	5.23 ± 0.06	0.045 ± 0.08	544.78 ± 1.90	0.01 ± 0.006	9.34 ± 0.8	0.11 ± 0.003
2A	9.30 ± 0.10	4.66 ± 0.02	0.033 ± 0.09	460.51 ± 0.01	0.02 ± 0.001	7.37 ± 0.05	0.08 ± 0.001
3A	10.22 ± 0.03	4.78 ± 0.02	0.130 ± 0.08	511.95 ± 1.90	0.01 ± 0.001	53.32 ± 0.52	0.05 ± 0.005
4A	12.56 ± 0.06	6.98 ± 0.02	0.065 ± 0.09	762.57 ± 0.02	0.43 ± 0.006	18.25 ± 0.06	0.10 ± 0.004
5A	6.60 ± 0.10	5.45 ± 0.04	0.050 ± 0.09	623.58 ± 1.90	0.07 ± 0.006	25.53 ± 0.18	0.12 ± 0.001
6A	9.23 ± 0.06	4.78 ± 0.006	0.063 ± 0.09	557.91 ± 1.90	0.01 ± 0.007	20.26 ± 0.07	0.09 ± 0.004
7A	12.13 ± 0.15	6.93 ± 0.006	0.130 ± 0.001	536.03 ± 0.01	0.05 ± 0.001	14.48 ± 0.04	0.04 ± 0.005
8A	19.00 ± 0.17	5.96 ± 0.12	0.077 ± 0.10	661.88 ± 1.90	0.36 ± 0.02	17.35 ± 0.03	0.12 ± 0.002
9A	5.66 ± 0.11	5.13 ± 0.006	0.373 ± 0.11	507.57 ± 3.79	0.19 ± 0.009	4.96 ± 0.06	0.14 ± 0.001
10A	24.66 ± 0.28	6.61 ± 0.05	0.123 ± 0.05	670.64 ± 0.001	0.02 ± 0.001	27.43 ± 0.08	0.09 ± 0.003
11A	12.96 ± 0.28	6.73 ± 0.006	0.119 ± 0.05	608.26 ± 0.002	0.41 ± 0.04	10.11 ± 0.07	0.13 ± 0.004
12A	5.80 ± 0.17	4.74 ± 0.02	0.048 ± 0.05	526.18 ± 0.001	0.03 ± 0.004	21.90 ± 0.02	0.09 ± 0.001
13A	5.20 ± 0.17	4.18 ± 0.05	0.038 ± 0.11	496.63 ± 0.001	0.14 ± 0.04	2.99 ± 0.03	0.09 ± 0.001
14A	6.50 ± 0.10	4.85 ± 0.03	0.022 ± 0.06	833.16 ± 0.001	0.01 ± 0.001	10.22 ± 0.12	0.08 ± 0.002
15A	10.86 ± 0.11	5.58 ± 0.06	0.168 ± 0.05	611.54 ± 0.001	0.05 ± 0.01	77.17 ± 0.14	0.08 ± 0.007
16A	12.80 ± 0.10	5.58 ± 0.11	0.113 ± 0.05	550.25 ± 1.90	0.22 ± 0.01	32.98 ± 0.27	0.09 ± 0.001

The content of the nitrogen forms in the three groups (autogenic, hydrogenic and lithogenic) of the C and A soils are presented in the Electronic Supplementary Material (Figs. S1 and S2, respectively). It should be stressed that the aforementioned N content refers to nitrogen pool that remains in soil after the former vegetation season (2013), as the soils were sampled before fertilisation (early spring 2014). It was found that the highest concentrations of the N forms were accumulated in the agriculturally exploited RL soils belonging to the lithogenic group (Fig. S1), which significantly differed from the two other groups with respect to NO_3_–N and NO_2_–N (*p* = 0.0000). Taken into account the NH_4_–N pool, no differences were noted between the lithogenic and hydrogenic soils; however, there was a significant difference between the autogenic and lithogenic soils (*p* = 0.001), where NH_4_–N reached an 80 % higher level in the lithogenic soils than in the autogenic (Fig. S1). The RL wasteland soils (Fig. S2) also contained significantly higher NO_3_–N content (c.a. by 43 %) than the autogenic and hydrogenic soils (*p* = 0.0023). There was a significant dominance of NO_2_–N (*p* = 0.0018) in the controls of the autogenic soils, as in the case of NH_4_–N, but the ammonium concentration did not differ significantly among the three soil groups (*p* = 0.2932, Fig. S2).

Deliberations concerning the N content in the different soil types are important from the point of prediction of the mineral fertilisation requirement. Thus, the effect of the land use on the content of the N form content in the autogenic (Fig. S3), hydrogenic (Fig. S4) and lithogenic soils (Fig. S5) was also investigated. In autogenic soils, represented by AL, BA and HP soils (Fig. S3), agricultural practices resulted in significant increase (c.a. by 60 %) of NO_3_–N (*p* = 0.0000) in comparison with controls and in 3-fold decrease of NO_2_–N (*p* = 0.0000) and NH_4_–N (*p* = 0.0000). Agricultural hydrogenic soils, represented by ML, EF and EH soil types (Fig. S4) also have higher pools of NO_3_–N and NH_4_–N than their corresponding controls; however, those differences were insignificant (*p* = 0.0684 and *p* = 0.0747, respectively). According to NO_2_–N concentrations, no differences was stated between A and C sites (*p* = 0.8284). Agricultural soil usage strongly affected lithogenic group, represented by RL soil type (Fig. S5) and resulted in higher (c.a. by 80 %) level of NO_3_–N (*p* = 0.0015) and NH_4_–N (*p* = 0.0432) than in controls, whereas NO_2_–N remained on similar level in agricultural and control sites (*p* = 0.0654).

DNA quantification is presented in Table S1 (see Electronic Supplementary Material). It was found that higher DNA content characterised C soils and amounted to 1.275–7.128 μg/g, whereas in A soils ranged from 0.834 to 3.835 μg/g. However, positive results of PCR were achieved independently on DNA content in every soil samples.

### Diversity of Potential Nitrogen-Fixing Bacteria in Autogenic, Hydrogenic and Lithogenic Soils

A total of 358,289 valid reads were obtained from 31 samples (together C and A soil samples) by Ion Torrent™ technology. Microbiome of A soils were represented by 21,366 OTUs classified as *Proteobacteria*, *Acidobacteria*, *Actinobacteria, Bacteroidetes*, *Elusimicrobia, Firmicutes*, *Chlorobi*, *Chloroflexi*, *Gemmatimonadetes*, *Planctomycetes*, *Spirochaeta* and *Verrucomicrobia*, whilst with respect to controls, higher by 6.07 % OTU number were noted and amounted to 22,664. However, by taking into account the number of *α*-*Proteobacteria*, *β*-*Proteobacteria* and *Cyanobacteria* OTUs, it was found that A soils contained 6625 identified OTUs whilst C soils 6902 OTUs, and among them, a total of 387–392 OTUs were classified as PNF bacteria, for C and A soils, respectively (Table 4). The highest number of PNF bacterial diversity (194–213 OTUs, for C and A) were noted in autogenic group of soils, then in hydrogenic (134–168 OTUs, for A and C) and the lowest in lithogenic soils (23–27 OTUs, for A and C variant).

**Table 4:** General number of *α*-*Proteobacteria*, *β*-*Proteobacteria* and *Cyanobacteria* OTUs and PNF bacteria in the three groups of control (C) and agricultural (A) soils

Phylogenetic group	OTU number					
	Lithogenic soil		Autogenic soil		Hydrogenic soil	
	C	A	C	A	C	A
*Proteobacteria*						
*α*-*Proteobacteria*	172^a^	474^a^	1720^a^	1906^a^	520^a^	557^a^
*Rhizobaceae*	5^a^	6^a^	22^a^	23^a^	35^a^	10^a^
*Rhizobium*	1^b^	2^b^	5^b^	5^b^	3^b^	2^b^
*Hyphomicrobiaceae*	16^a^	10^a^	62^a^	69^a^	110^a^	24^a^
*Devosia*	4^b^	3^b^	16^b^	16^b^	9^b^	7^b^
*Methylobacteraceae*	1^a^	1^a^	2^a^	4^a^	5^a^	3^a^
*Microvirga*	1^b^	1^b^	1^b^	4^b^	2^b^	2^b^
*Methylobacterium*	0^b^	0^b^	1^b^	0^b^	3^b^	1^b^
*Phyllobacteraceae*	4^a^	3^a^	9^a^	13^a^	18^a^	4^a^
*Mesorhizobium*	2^b^	1^b^	8^b^	9^b^	4^b^	4^b^
*Phyllobacterium*	0^b^	0^b^	0^b^	0^b^	1^b^	0^b^
*β*-*Proteobacteria*	335^a^	768^a^	2527^a^	2765^a^	1062^a^	756^a^
*Burkholderiaceae*	13^b^	8^b^	92^b^	97^b^	114^b^	74^b^
*Burkholderia*	5^b^	4^b^	48^b^	54^b^	23^b^	26^b^
*Cupriavidus*	0^b^	2^b^	8^b^	11^b^	7^b^	4^b^
*Cyanobacteria*	1^b^	2^b^	15^b^	17^b^	2^b^	14^b^

^a^General number of OTUs noted in the current study

^b^OTUs dedicated for PNF bacteria

In general, the investigated Polish arable soils were decidedly dominated by *β*-*Proteobacteria* representatives of PNF bacteria (183 and 193 OTUs, for C and A, respectively). In this class, *Burkholderiaceae* family and *Burkholderia* genus were the most important (Table 4). Representatives of *Burkholderia* usually preferred A sites belonging to autogenic and hydrogenic soils (54 and 26 OTUs, respectively). Quite high *Burkholderia* numbers were also found in C sites (48 and 23 OTUs), assigned for the following autogenic and hydrogenic soils. In contrary, lithogenic soils constituted the least-preferred niches for *Burkholderia* inhabitants (4–5 OTUs). Subdominants of *β*-*Proteobacteria* were *Cupriavidus* representatives which demonstrated similar to *Burkholderia* preferences for niche occupation (Table 4).

Among PNF bacterial OTUs assigned to *α*-*Proteobacteria*, the domination of *Hyphomicrobiaceae* representatives was noted and among them *Devosia* were the most abundant (16 OTUs in autogenic soils, 7–9 OTUs in hydrogenic and 3–4 OTUs in lithogenic soils). Subdominants were *Rhizobiaceae* and *Phyllobacteriaceae* families with *Rhizobium*, *Mesorhizobium* and *Phyllobacterium* representatives, respectively. Generally, among each of the main PNF phyla, higher OTU number was noted in autogenic rather than in hydrogenic and finally lithogenic groups of soils, which suggests that soil formation process is a conducive factor for PNF bacteria preference for soil niche occupation.

**Fig. 2:**
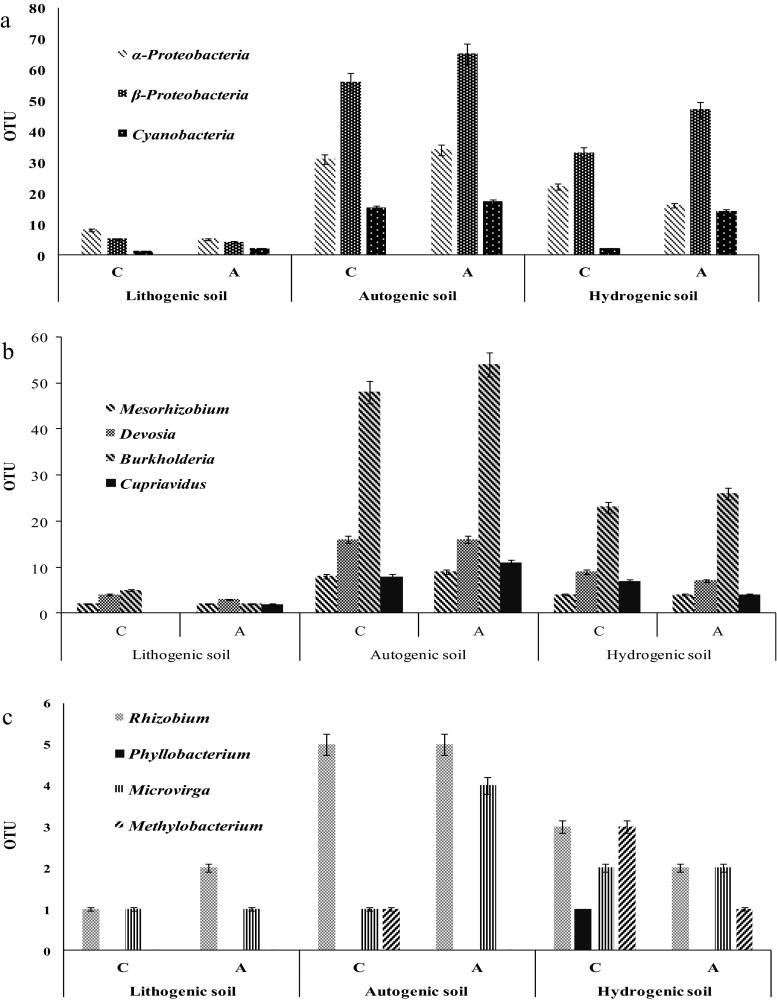
The main phyla of PNF bacteria (**a**), dominant *Proteobacteria* OTUs (**b**) and subdominant *Proteobacteria* OTUs (**c**) in the three groups of control (c) and agricultural (a) soils. Mean values with standard error (SE) are presented

As presented in Fig. 2a, the PNF bacterial community structure depended both on soil formation process as on the way of land use. Anyhow, from 31 investigated sites (A and C), eight important genera of PNF bacteria were identified, among which *Devosia*, *Mesorhizobium*, *Burkholderia* and *Cupriavidus* were classified according to OTU numbers as dominant PNF bacteria (Fig. 2b). The same, those genera with OTUs <10 were characterised as subdominants of PNF bacteria in Polish soils (Fig. 2c). Usually, OTU numbers were higher in A soils in respect to *Burkholderia*, *Cupriavidus*, *Mesorhizobium* and *Microvirga* or remained on similar level with C soils with respect to *Mesorhizobium* and *Rhizobium* representatives. Significant decrease of OTUs number as a consequence of soil agricultural exploitation was noted in relation to *Devosia* and *Methylobacterium*, whereas the presence of *Phyllobacterium* was stated only in C soils.

As was earlier mentioned the seven investigated soil types were classified into the three groups according to their origin (bedrock formation): lithogenic, autogenic and hydrogenic. Our result clearly demonstrated that not only way of land use (cultivation or non-cultivation) but also soil formation process is an important factor for subsequent microbial PNF bacteria diversity colonisation preferences. Decidedly, the highest number of PNF bacteria were found in autogenic group represented by AL, BA and HP soil types, with domination of *Proteobacteria* (87 and 99 OTUs for C and A soils, respectively) and among them *Burkholderia* genus (48 and 54 OTUs). Likewise, the number of *Cyanobacteria* were the highest in autogenic A soils (17 OTUs) than C sites (15 OTUs). This trend was also supported with respect to subdominant PNF bacterial OTUs that also reached the highest abundance in autogenic soils with considerable (c.a. 20 %) surplus of *Rhizobium* on the background of other identified genera (Fig. 2c). *Microvirga* representatives subdominated in the agricultural autogenic soils whilst in controls its abundance decreased by 25 %. On the contrary, C autogenic soils contained representatives of *Methylobacterium* whilst in A soils mentioned genus was not detected.

The microbiome of hydrogenic soils, represented by MG, EF and EH soil types (both C and A) were definitely lower than those of autogenic soils, anyhow also *Proteobacteria* dominance was stated (52 and 46 OTUs for C and A sites) with predomination of *Burkholderia* (23 and 26 OTUs). Subdominant OTUs of PNF bacteria in C hydrogenic soils were formed by *Rhizobium*, *Methylobacterium*, *Microvirga* and *Phyllobacterium* whilst in A soils mentioned OTU number were reduced by c.a. 30 and 66 % for *Rhizobium* and *Methylobacterium*, respectively. *Microvirga* OTU number remained on the same level regardless of the way the soil was used, whereas *Phyllobacterium* was not present in agricultural hydrogenic soils.

However, the lowest level of PNF bacterial community abundance were observed with respect to lithogenic soils, represented by RL soil type, which testifies that those soil categories are not optimal niches for PNF bacteria development. *Rhizobium* population was reduced by 20–40 % than its OTU number noted in hydrogenic or autogenic soils, respectively, whereas *Microvirga* remained on the same level both on C and A soils. It should be underlined that in the lithogenic group of soil, the presence of *Methylobacterium* and *Phyllobacterium* has not been detected. In order to determine the preferable conditions for living in soil environment of PNF bacteria, the relationships among soil features and bacterial OTUs were tested.

### Ecological Characteristic of Potential Niches for Nitrogen-Fixing Bacteria

The significant relationships between pH and the bacterial community of PNF have been demonstrated in all type of soils. It was found that the abundance of *Burkholderia* significantly decreased with the acidic pH, below 5.5 (respectively LG: *p* < 0.05, *ρ* = −0.196; AG: *p* < 0.05, *ρ* = −0.538; and HG: *p* < 0.05, *ρ* = −0.852; Table 5), which suggests that PNF bacteria prefer rather higher pH values, close to the neutral conditions or even alkaline than acidic. The same phenomenon has been shown in relation to the abundance of *Cupriavidus*. The negative significant correlation between pH and the number of OTUs were found in the AG and LG soils (LG: *p* < 0.05, *ρ* = −0.361; AG: *p* < 0.05, *ρ* = −0.471). The situation is unlike in the HG soils, where the positive correlation between pH and the number of OTUs (*p* < 0.05, *ρ* = 0.219) were demonstrated. Probably, this is connected with the naturally low pH of the HG soils (Table 3). No correlation between pH and the abundance of *Mesorhizobium* was found, except for the LG soils (*p* < 0.05, *ρ* = 0.398). Soil pH also indicates some negative effects on the abundance of *Cyanobacteria* (LG: *p* < 0.05, *ρ* = −0.994; AG: *p* < 0.05, *ρ* = −0.441; HG: *p* < 0.05, *ρ* = −0.466). The pH of these soils was slightly acidic (c.a. 5.51), and much lower than the optimum pH (7–10).

**Table 5:** Correlations among investigated soils features and PNF bacteria OTUs with respect to lithogenic (LG), autogenic (AG) and hydrogenic (HG) soils (*n* = 32, *p* < 0.05)

Factor	OTU number																					
	*Burkholderia*			*Cupriavidus*			*Mesorhizobium*		*Devosia*			*Rhizobium*			*Methylobacterium*		*Microvirga*			*Cyanobacteria*		
	LG	AG	HG	LG	AG	HG	LG	AG/HG	LG	AG	HG	LG	AG	HG	LG/AG	HG	LG	AG	HG	LG	AG	HG
MOIST (%)	ns	−0.462	−0.954	ns	0.215	ns	ns	0.341/ns	ns	0.109	0.963	ns	0.120	0.405	ns/-0.287	−0.963	ns	ns	0.773	ns	−0.414	−0.483
pH	−0.196	−0.538	−0.852	−0.361	−0.471	0.219	0.398	ns	0.398	ns	ns	0.548	ns	ns	ns	−0.243	0.548	ns	0.629	−0.994	−0.441	−0.466
EDC (mg kg^−1^)	ns	−0.389	−0.657	−0.342	−0.416	ns	0.506	ns	0.506	−0.255	0.585	0.545	ns	−0.211	ns	−0.206	0.545	ns	ns	−0.972	−0.284	−0.684
EC (mS cm^−1^)	−0.2636	0.389	−0.223	0.844	0.424	ns	0.331	ns	0.331	ns	−0.664	0.886	0.403	−0.342	ns	−0.406	0.886	ns	0.614	ns	0.263	ns
NO_2_–N (mg kg^−1^)	ns	−0.306	−0.441	0.998	−0.301	0.509	0.998	ns	0.366	ns	−0.222	0.620	ns	ns	ns	−0.262	0.620	ns	0.379	0.283	ns	−0.236
NO_3_–N (mg kg^−1^)	ns	ns	ns	0.946	0.407	−0.394	ns	ns	ns	ns	ns	0.449	−0.360	−0.658	ns	−0.689	0.449	ns	0.536	0.427	ns	0.693
NH_4_–N (mg kg^−1^)	ns	ns	ns	ns	0.998	ns	0.316	ns	0.316	−0.407	ns	0.610	−0.293	−0.300	ns	−0.451	0.610	ns	0.453	0.294	ns	0.912

*ns* no significance

Also, soil moisture influenced the PNF bacteria community (Table 5). Positive correlations were noted between MOIST and OTU number inhabiting AG soils and belonging to the following genera: *Cupriavidius* (*p* < 0.05, *ρ* = 0.215), *Mesorhizobium* (*p* < 0.05, *ρ* = 0.341), *Devosia* (*p* < 0.05, *ρ* = −0.109) and *Rhizobium* (*p* < 0.05, *ρ* = 0.120). The last two displayed positive relationship also with respect to HG soils (*p* < 0.05, *ρ* = 0.963 and *ρ* = 0.405), respectively. The increase of MOIST was also optimal to *Microvirga*-inhabiting HG soils (*p* < 0.05, *ρ* = 0.773. However, *Burkholderia*, *Methylobacterium* and *Cyanobacteria* being present in AG and HG soils preferred rather dry soil conditions and displayed negative correlations with MOIST as presented in Table 5.

Carbon (especially its easily available form) was an important limitation factor for PNF bacterial growth. We obtained significant negative correlation between EDC and OTU number with respect to *Burkholderia*, *Cupriavidus*, *Devosia* and *Rhizobium* (*p* < 0.05, *ρ* = −0.2199 and *p* < 0.05, *ρ* = −0.397), which means that those PNF bacteria are the most sensitive on EDC supply. A similar trend was observed for the abundance of *Cyanobacteria* and EDC value (*p* < 0.05; LG: *ρ* = −0.994; AG: *ρ* = −0.284; HG: *ρ* = −0.684), which indicated that the microorganisms belonging to this phylum are significantly EDC dependent. Moreover, it seems that EC can considerably modulate the OTU number of PNF bacteria. We showed a positively correlated EC value and the number of OTU for *Burkholderia* (only for AG soils, *p* < 0.05, LG: *ρ* = 0.389), *Cupriavidus*, *Mesorhizobium*, *Devosia* (only for LG soils, respectively, *p* < 0.05; LG: *ρ* = −0.994, *ρ* = −0.331, *ρ* = −0.331), *Rhizobium*, *Microvirga* and *Cyanobacteria*.

The nitrogen content (NO_2_–N, NO_3_–N, NH_4_–N) has a different impact on the number of PNF bacteria OTUs. It was shown that there are positive correlation between the abundance of *Microvirga* in soils and the nitrogen content (Table 5). The OTU number of *Cyanobacteria* can also be modified by their NO_2_–N, NO_3_–N and NH_4_–N contents (Table 5).

## Discussion

To date, majority of studies related to nitrogen-fixing bacteria were concentrated on symbiosis between them and different legumes [2, 26]. Anyhow, there is a lack of knowledge about PNF bacterial groups, inhabiting agricultural soils, which may not be under symbiosis relation with legumes but are present in the soil environment and able to perform potential nitrogen fixation process. Moreover, the novelty of our study is to demonstrate that soil formation process is a crucial factor for PNF bacterial diversity in with regard to Polish agricultural and control soils. Here, we analysed the biodiversity of PNF bacteria with application of NGS technique through which the shortage and limitation connected with detecting only the most abundant and cultivated genera were omitted. Besides precise recognition of PNF bacterial diversity in agricultural soils and wastelands, optimal niche conditions were also determined.

We found the operational taxonomic units assigned to free-living (*Cyanobacteria*) and potential plant-associated nitrogen-fixing bacteria (*Burkholderia*, *Devosia*, *Cupriavidus* and *Rhizobium*). It should be noted that OTUs are a proxy for potential bacterial activity but do not reveal the nature of the bacterial activity. Presumably, the high relative abundance belonging to the PNF bacteria can indicate that the activity of nitrogen-fixing bacteria may be of ecological importance at the study site, especially in agricultural soil.

Determination of N contents in agricultural soils in spring is a useful tool to assess requirements for nitrogen fertilisation, whilst the distribution of NO_3_–N in soil in autumn evaluates possible nitrogen losses by leaching during winter [16]. It was also assumed that soil agricultural practices influence the activity of microorganisms which participate in various nitrogen transformation processes in soil [45]. Importantly, our results reflect similarity of soil science classification with respect to soil formation processes and microbial biodiversity evolution and colonisation preferences. It seems that the lithogenic soils with lowest abundance of PNF revealed their need for mineral fertilisation by adding additional nitrogen compounds. Although this soil group contained the highest N pool (remaining after last vegetation season) among each investigated soils (Figs. S1 and S5), it turned out to be the most requiring and most sensitive of N content, and thus rational fertilisation of RL soils is reasonable. Moreover, the obtained results suggested that the autogenic and hydrogenic soils have enough abundance of PNF, especially when considering lack of significant differences in the number of OTUs between agricultural and control soils.

The dominant *Burkholderia* genus achieved higher abundance by 70 and 87 % in hydrogenic and autogenic soils, respectively, than in lithogenic soils. The same abundance of *Devosia* was by 55 and 70 % higher for hydrogenic and autogenic soils with regard to lithogenic. Thus, it may be suspected that both autogenic as hydrogenic soils do not require additional fertilisation because they are able to use the N resources accumulated in the ground and this N pool is sufficient for inhabiting PNF bacteria. Furthermore, the results from Wood et al.’s [46] study suggest that soil bacterial diversity decreased with mineral fertiliser addition. These authors recommended fertilisation by combining mineral fertilisers with organic inputs. It seems that this fertilisation strategy is appropriate for Polish lithogenic soils. The suitable fertilisation and another way of land use as well as concern for beneficial soil microflora may have an impact in soil ecosystem functionality, especially with respect to agriculture. Comparable results were demonstrated by Montecchia et al. [47], who studied changes in soil bacterial communities after conversion of the Yungas forests to agriculture. Their results indicated that communities with many taxa (especially the relative abundance of copiotrophic and oligotrophic taxa) may have many functional attributes, allowing to maintain at least some soil ecosystem services after forest conversion to croplands.

It was assumed that the PNF bacteria population could be affected by a number of different chemical and biological factors. Among them, impact of pH [48], soil moisture [49], carbon and nitrogen content [1, 50], EC [50] and total phosphorus [51] are the most known and described. Significant impact of pH, moisture, carbon and nitrogen concentrations and EC were also demonstrated in the current study. Bartram et al. [48] reported that bacterial diversity is the lowest in acidic pH and displayed increasing trend with increase pH values. They also determined optimal pH (7.5) for maintaining biodiversity. However, there are some species of bacteria for which pH is not a limiting factor. One of them is bacteria belonging to *Mesorhizobium*. In the literature, strains which can grow at pH values ranging between 4.5 and 10.5, for example *Mesorhizobium shonense* [52] are known. The analogical situations exist for the abundance of *Devosia* and *Rhizobium*. For *Methylobacterium*, optimal growth conditions are reported to be close to neutrality, although some strains can grow at pH 4 and some at pH 10. Therefore, our results indicate only a correlation between pH of HG soils and the abundance of *Methylobacterium*.

Obtained results were compared with those of other researchers and summarised in Table 6. Investigated Polish soils were characterised by the lowest pH values which in the case of arable soils was acidic (pH 5.51) and one unit higher (pH 6.15) with respect to wastelands used as controls. As the impact of pH on PNF bacteria is known to be the most significant, global soils were classified according to pH value, dominant phyla and predominant genera of nitrogen-fixing bacteria. In those conditions, populations of PNF bacteria were classified as *Proteobacteria* phylum (*α*- and *β*-classes*)* with domination of the *Burkholderia* genus both in A and C soils. By comparison, the pH values of other global soils stated that those from Antarctica region had alkaline pH (8.5–9.9) and the dominant phylum was *Cyanobacteria* and *Proteobacteria* with *Geobacter* as predominant genus [53]. Soils with pH close to neutral (6.7) from the Arctic tundra and scrubland soils were dominated by *Rhodopseudomonas* genus [54, 55], whereas those with pH higher than 7 by *Methylocella* [56] and *Bradyrhizobium* [25] for Tibetan prairie soil and Quilian meadows, respectively.

**Table 6:** Well recognised nitrogen-fixing bacterial communities in different region in the world

Study regions	Average soil pH	Dominant phyla	Predominant genera	Reference
Antarctica-wetted soil	8.48	*α-Proteobacteria*	*Geobacter*	[53]
		*β-Proteobacteria*		
		*γ-Proteobacteria*		
		*δ-Proteobacteria*		
		*Cyanobacteria*		
Antarctica microbial mat	9.90	*β-Proteobacteria*	*Azotobacter*	[68]
		*γ-Proteobacteria*		
		*δ-Proteobacteria*		
		*Firmicutes*		
		*Spirochaetes*		
		*Cyanobacteria*		
		*Verrucomicrobia*		
		Unidentified cluster		
Canadian Arctic scrubland soil	–	*α-Proteobacteria*	*Rhodopseudomonas*	[54]
		*β-Proteobacteria*		
		*γ-Proteobacteria*		
		*Firmicutes*		
		Unidentified cluster		
Arctic tundra soil	6.70	*α-Proteobacteria*	*Rhodopseudomonas*	[55]
		*γ-Proteobacteria*		
		*δ-Proteobacteria*		
		*Spirochaetes*		
		*Cyanobacteria*		
		*Spirochaetes*		
		Unidentified cluster		
Tibetan plateau alpine prairie soil	7.43	*α-Proteobacteria*	*Methylocella*	[56]
		*β-Proteobacteria*		
		*γ-Proteobacteria*		
		*δ-Proteobacteria*		
		*Unidentified cluster*		
Qilian mountains scrubland and meadow soil	7.04	*α-Proteobacteria*	*Bradyrhizobium*	[25]
		*β-Proteobacteria*		
		*γ-Proteobacteria*		
		Unidentified cluster		
Polish agriculture soils	5.51	*α-Proteobacteria*	*Burkholderia*	This study
		*β-Proteobacteria*		
Polish wastelands soils	6.15	*α-Proteobacteria*	*Burkholderia*	
		*β-Proteobacteria*		

Domination of *Bradyrhizobium* with respect to Amazon soils under agroforestry system (Cowpea grain-producing legume) was reported by Jaramillo et al. [10]. Specific nitrogen-fixing microbiome of switchgrass that is native to the tallgrass prairies of North America were represented by *Rhizobium* and *Methylobacterium* species of the *α*-*Proteobacteria*, *Burkholderia* and *Azoarcus* species of the *β*-*Proteobacteria* and *Desulfuromonas* and *Geobacter* species of the *δ-Proteobacteria* [3].

The genus *Burkholderia*, the members of the *β*-*Proteobacteria* class in the context of results from the current study deserve the most attention. This bacterial group was reported to contain most of all species that nodulate legumes; however, several families are included also in *α*-*Proteobacteria*, i.e. *Methylobacterium* [57]. What is more, *Burkholderia* genus are known to be versatile organisms that occupy a wide range of ecological niches [24, 58], i.e. soil (also contaminated), water (also sea water), rhizosphere, humans and hospital environment. This testifies that those bacterial groups are resistant to different stresses and possess quick ability for adaptation in different environments. The current study demonstrated its domination in agricultural soils rather than in wastelands. The genus *Burkholderia* comprises over 60 species; majority of which, were verified as an effective nitrogen fixers [22, 58], with *B. cepacia* at the forefront [59].

Unfortunately, data above presented advantages that some *Burkholderia* species have been involved in human and plants infections and classified as pathogens [24]. Fortunately, the majority of *Burkholderia* species are known as soil bacteria, exhibited rather non-pathogenic interactions with plants, i.e. they promote plant growth, can degrade the organic compounds of anthropogenic origin and/or may result in biocontrol of pathogens [24, 58, 59]. *Burkholderia* species also have the potential to be used as plant-growth-promoting rhizobacteria as some mechanisms to promote plant growth in this genus were detected [59]. This fact is important for a potential use of *Burkholderia* in agriculture. The first known diazotroph was *Burkholderia vietnamiensis* isolated from the rhizosphere of young rice seedlings in Vietnam [60]. It was also reported that soil inoculation with *Burkholderia brasiliense* and *B. vietnamiensis* resulted in 42–64 % increase in growth of rice plants [61]. Moreover, endophytes of *Burkholderia* occurring in the Brazilian rice roots, steam and leafs are able to fix 31 % of N that protect the rice plantation and stimulate even by 69 % rice crop when compared with the control condition [61].

In relation to *Devosia*, the significant decrease of OTU number in comparison with other microbiota was observed. The genus *Devosia* was created from the reclassification of *Pseudomonas riboflavia* as *Devosia riboflavia* [62] and comprised eight well-recognised species [63]. Among these, only *D. neptuniae* was isolated from an aquatic leguminous plant and reported to possess the nitrogen-fixing (*nifH*) and the nodulating (*nodD*) symbiotic genes [64, 65].

We also found in Polish arable soils a high relative abundance of 16S rRNA sequences assigned to free-living *Cyanobacteria*, which suggest that their activity may be of ecological importance at the study site. Vijayan and Ray [66] indicated that there are positive correlations on the total number of *Cyanobacteria* inhabiting tropical paddy wetland to total nitrogen in these soils. Wakelin et al. [1] assumed that N_2_ fixed by those bacterial group is important in providing N pool necessary to support the decomposition of crop residues usually characterised by a wide C/N ratio. Chouhan and Kumawat [67] isolated six strains of *Cyanobacteria* from agricultural and grassland soils, which were assigned to the following genera: *Oscillatoria*, *Fischerella*, *Nostoc*, *Synechocystis* and *Gloeocapsa*. The obtained results indicated that *Cyanobacteria* belonging to the genera *Fischerella* and *Nostoc* can be used in biofertiliser production to improve agriculture and grassland soil fertility. What is more, they also provide nitrogen to plants and other organisms and are able to survive in both wet and dry conditions [67].

Our results also indicate that PNF bacteria may be sensitive on agricultural practices as well as could show resistance in response to agricultural way of land use and consequently remain insensitive. The PNF bacteria that potentially inhabit the LG and HG soils seemed to be definitely sensitive on agricultural operations as decrease of its OTU number in A soils was observed (Table 7). However, we demonstrated that microorganisms belonging to *β*-*Proteobacteria* in all studied soils displayed resistance to agricultural way of land use; therefore, there are the dominant group of PNF microorganisms in tested soils (Table 6). What is more, the abundance of *β*-*Proteobacteria* is higher in agricultural than control soils. Our results also demonstrated that agricultural operations had positive impact on the *Cyanobacteria* populations as we observed increasing trend in OTU number classified to *Cyanobacteria* in A soils, especially from HG group (Table 7).

**Table 7:** The number of PNF bacteria OTUs in the three groups of control (C) and agricultural (A) soils

	Lithogenic soils		Autogenic soils		Hydrogenic soils	
	C	A	C	A	C	A
*Proteobacteria*	13	9	87	99	55	63
α-*Proteobacteria*	8	5	31	34	22	16
β-*Proteobacteria*	5	4	56	65	33	47
*Cyanobacteria*	1	2	15	17	2	14

In summary, these results demonstrated that Polish arable soils are decidedly dominated by PNF bacteria from the *β*-*Proteobacteria* class and *Burkholderia* genus. Subdominants are bacteria of *α*-*Proteobacteria* class and *Devosia* genus. *Cyanobacteria* population dominated in agricultural rather than in control soils. PNF bacteria classified as rare OTUs were represented by the genera of *Rhizobium*, *Microvirga*, *Methylobacterium* and *Phyllobacterium*. Their abundance was directly connected with the soil formation process as most of them inhabit the autogenic group of soils formed on the loess material whilst the lowest PNF bacteria number was noted in the lithogenic soils, formed on limestone. What is more, in the lithogenic soil biodiversity of PNF bacteria was menacingly limited as lack of *Cupriavidus*, *Methylobacterium* and *Phyllobacterium* genera was stated. We also demonstrated that lithogenic soils demand additional fertiliser application as they seemed to be the most requiring and sensitive on N pool in the ground, in contrary to autogenic and hydrogenic soils. Determined optimal niche conditions preferable by PNF bacteria are as follows: neutral or alkaline pH, EC on the level at least 0.05–0.08 mS cm^−3^, and EDC >1300 mg kg^−1^; otherwise, it is a limiting factor for PNF growth. Effect posed by NO_3_–N remain unrecognisable and demand more studies as we observed its positive effect with regard to *Microvirga*, *Cyanobacteria* in LG soils, *Cupriavidus* in AG and HG soils, neutral with respect to *Burkholderia*, *Mesorhizobium*, *Devosia* and *Methylobacterium* in AG soils, and negative in relation to *Rhizobium* in AG and HG soils, *Methylobacterium* in HG soils and *Cupriavidus* in HG soils.

## Electronic Supplementary Material

Below is the link to the electronic supplementary material.

ESM 1 (DOCX 400 kb)
