# Supplementary material for: Metagenomic Analysis of Some Potential Nitrogen-Fixing Bacteria in Arable Soils at Different Formation Processes
Source: Microb Ecol. 2016 Aug 31;73(1):162–76. doi: 10.1007/s00248-016-0837-2 (PMC5209426; doi:10.1007/s00248-016-0837-2)
Supplement: Supplementary file 1 — (DOCX 400 kb) [file 248_2016_837_MOESM1_ESM.docx]

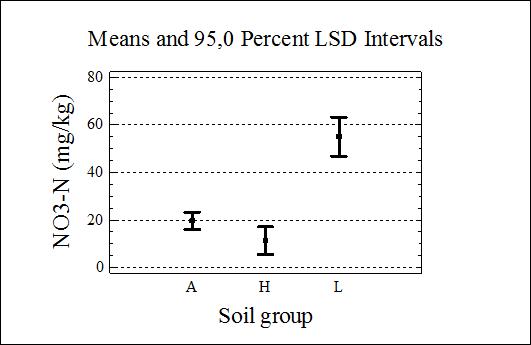


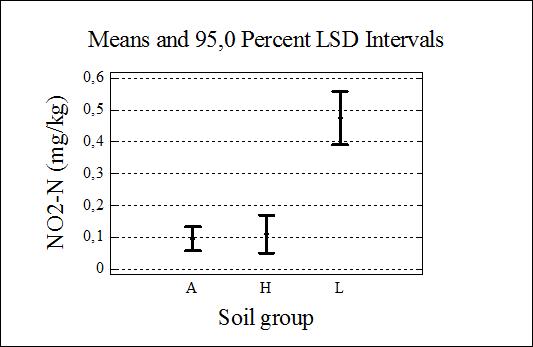


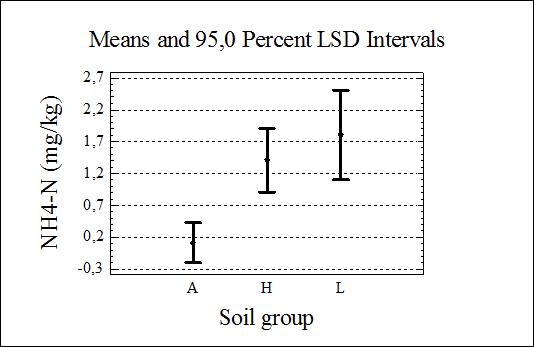


**Fig. 1S** Nitrogen form content in the three group of agricultural soils (n=84).

A - autogenic; H - hydrogenic, L - lithogenic


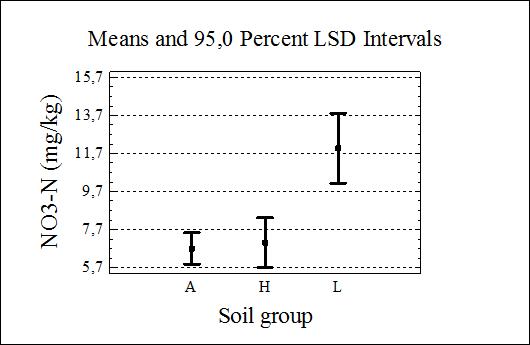


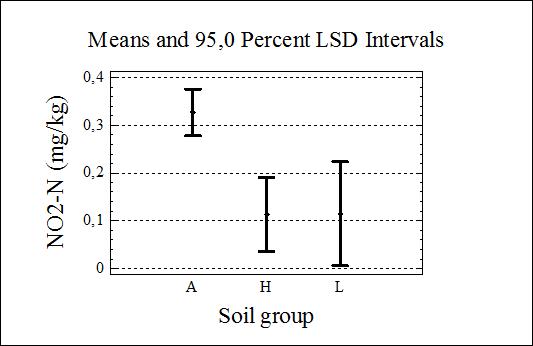


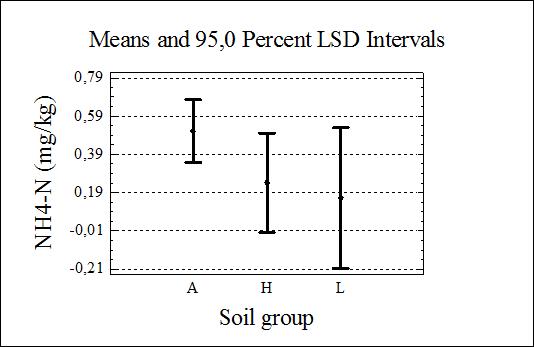


**Fig. 2S** Nitrogen form content in the three group of control soils (wastelands), n=84.

A - autogenic; H - hydrogenic, L - lithogenic


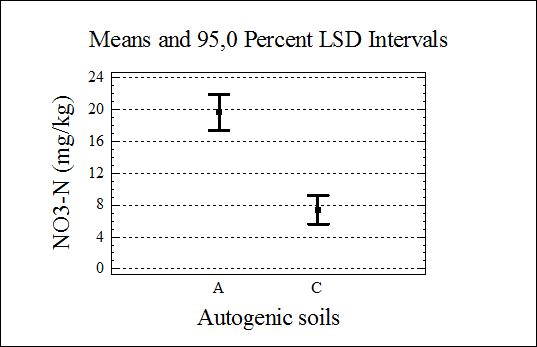


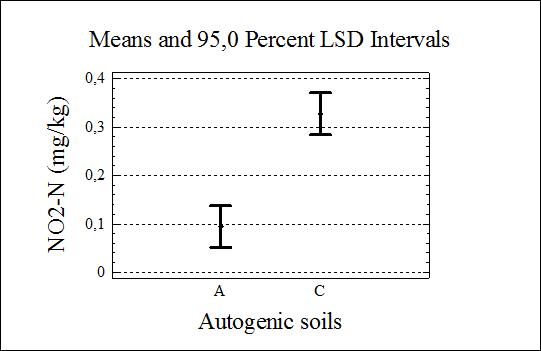


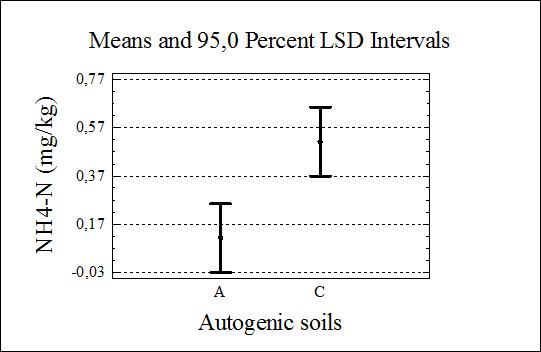


**Fig. 3S** Land use effect on nitrogen forms content in autogenic soils (n=60).

A - agricultural soils; C - controls (wastelands)


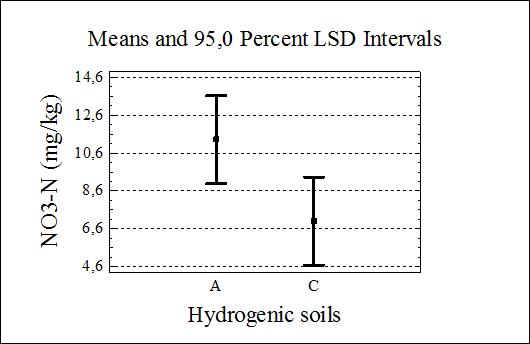


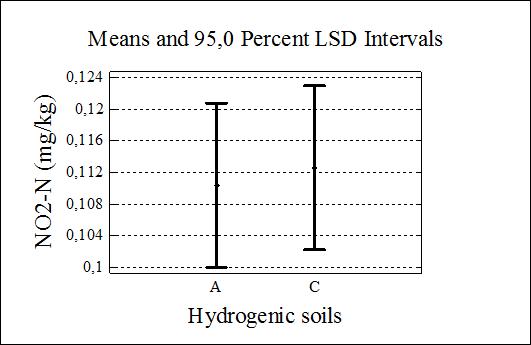


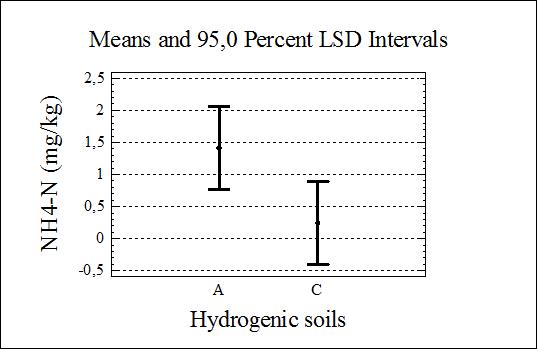


**Fig. 4S** Land use effect on nitrogen forms content in hydrogenic soils (n=24).

A - agricultural soils; C - controls (wastelands)


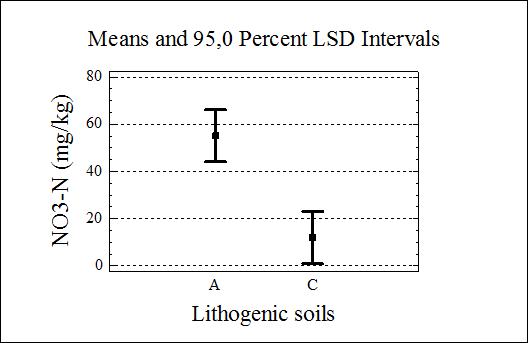


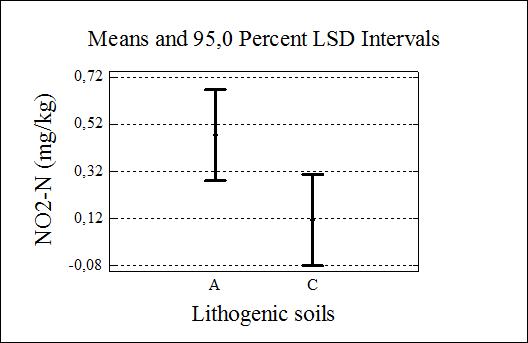


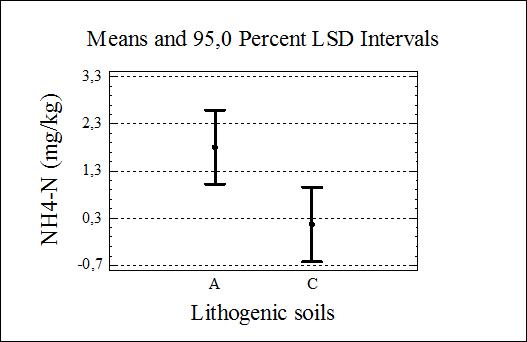


**Fig. 5S** Land use effect on nitrogen forms content in lithogenic soils (n=12).

A - agricultural soils; C - controls (wastelands)

**Table 1S** DNA concentration and purity coefficient (A_260_/A_280_) in agricultural (A) and control (C) soils

| **Agricultural**  **soils** | **DNA [µg/g]** | **A_260_/A_280_** | **Control soils** | **DNA [µg/g]** | **A_260_/A_280_** |
| --- | --- | --- | --- | --- | --- |
| 1A | **0.950** | **1.88** | 1C | **1.876** | **1.99** |
| 2A | **3.650** | **1.87** | 2C | **3.676** | **1.93** |
| 3A | **0.834** | **1.57** | 3C | **1.275** | **1.68** |
| 4A | **3.835** | **1.82** | 4C | **3.611** | **1.90** |
| 5A | **0.585** | **1.91** | 5C | **7.128** | **1.75** |
| 6A | **2.291** | **1.77** | 6C | **7.128** | **1.75** |
| 7A | **0.779** | **2.37** | 7C | **2.362** | **1.93** |
| 8A | **1.383** | **1.87** | 8C | **4.396** | **1.80** |
| 9A | **1.414** | **1.67** | 9C | **3.664** | **1.74** |
| 10A | **0.263** | **1.26** | 10C | **2.411** | **1.85** |
| 11A | **2.946** | **1.86** | 11C | **3.992** | **1.82** |
| 12A | **3.137** | **1.91** | 12C | **1.700** | **1.59** |
| 13A | **0.979** | **1.74** | 13C | **1.546** | **2.10** |
| 14A | **1.227** | **1.68** | 14C | **2.726** | **1.82** |
| 15A | **2.715** | **1.71** | 15C | **3.428** | **1.73** |
| 16A | **2.069** | **1.77** | 16C | **3.448** | **1.86** |
